# Insulin-dependent GLUT4 trafficking is not regulated by protein SUMOylation in L6 myocytes

**DOI:** 10.1038/s41598-019-42574-3

**Published:** 2019-04-24

**Authors:** Ruth E. Carmichael, Kevin A. Wilkinson, Tim J. Craig

**Affiliations:** 1 Centre for Research in Biosciences, University of the West of England, Coldharbour Lane, Frenchay, BS16 1QY UK; 2 School of Biochemistry, Biomedical Sciences Building, University of Bristol, University Walk, Bristol, BS8 1TD UK; 3 College of Life and Environmental Sciences, Geoffrey Pope Building, University of Exeter, Stocker Road, Exeter EX4 4QD, Exeter, United Kingdom

**Keywords:** Insulin signalling, Sumoylation

## Abstract

Type-II Diabetes Mellitus (T2DM) is one of the fastest growing public health issues today, consuming 12% of worldwide health budgets and affecting an estimated 400 million people. One of the key pathological traits of this disease is insulin resistance at ‘glucose sink’ tissues (mostly skeletal muscle), and this remains one of the features of this disease most intractable to therapeutic intervention. Several lines of evidence have implicated the post-translational modification, SUMOylation, in insulin signalling and insulin resistance in skeletal muscle. In this study, we examined this possibility by manipulation of cellular SUMOylation levels using multiple different tools, and assaying the effect on insulin-stimulated GLUT4 surface expression in differentiated L6 rat myocytes. Although insulin stimulation of L6 myocytes produced a robust decrease in total cellular SUMO1-ylation levels, manipulating cellular SUMOylation had no effect on insulin-responsive GLUT4 surface trafficking using any of the tools we employed. Whilst we cannot totally exclude the possibility that SUMOylation plays a role in the insulin signalling pathway in human health and disease, our data strongly argue that GLUT4 trafficking in response to insulin is not regulated by protein SUMOylation, and that SUMOylation does not therefore represent a viable therapeutic target for the treatment of insulin resistance.

## Introduction

Type-II Diabetes Mellitus (T2DM) is one of the most pressing public health concerns of modern times, with alarming increases in incidence shown in every country for which reliable records exist. The increase in T2DM is most stark in rapidly developing economies, and closely associated with a transition to a highly calorific, western-style diet^1^. T2DM is a chronic and generally progressive failure of blood glucose homeostasis, resulting in hyperglycaemia and many serious complications, including increased risk of cardiovascular disease, diabetic retinopathy, kidney failure and lower limb amputations. Globally, the treatment and management of T2DM consumes over 12% of the total health expenditure, and this figure is predicted to rise^2^. Despite the gravity of this situation, much is still not understood about the pathogenesis of T2DM, in part due to the largely asymptomatic presentation of the prediabetic state. However, the main pathological defects in T2DM are generally accepted to be a lack of insulin secretion from pancreatic beta cells^3^ and insulin resistance at insulin-responsive tissues^4^, e.g. liver, muscle and fat.

**Figure 1:**
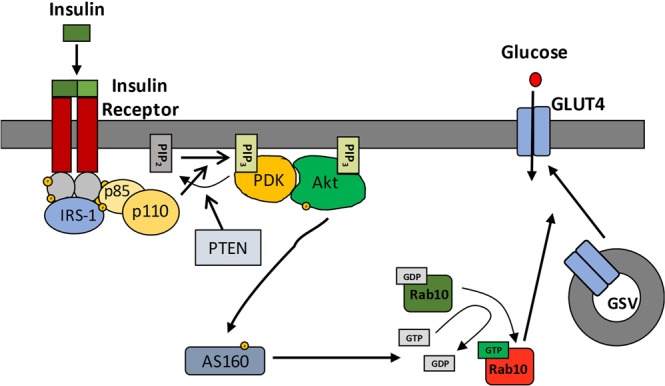
Schematic of the Insulin Signalling Pathway and insulin resistance. The insulin receptor is a receptor tyrosine kinase, which undergoes dimerisation and autophosphorylation on insulin binding. The phosphorylated receptor recruits and phosphorylates the insulin receptor substrate 1 (IRS-1) on tyrosine residues, which then recruits dimeric PI3 kinase via SH2 domains on the p85 subunit. PI3 kinase catalyses the phosphorylation of phosphatidylinositol bisphosphate (PIP_2_) at the plasma membrane to PIP_3_ (reversed by PTEN), which then recruits PIP3-dependent kinase (PDK) and Akt, allowing PDK to phosphorylate and activate Akt. Activated Akt phosphorylates and inactivates the Rab10 GAP, AS160, allowing sustained Rab10 activation which plays a critical role in trafficking of GLUT4 storage vesicles (GSVs) to the plasma membrane and surface expression of GLUT4. High levels of free fatty acids lead to an accumulation of lipid-derived second messengers, e.g. diacylglycerol and ceramide, which can inhibit the pathway at several different stages.

Insulin is a critical hormone for the regulation of blood glucose levels. After ingestion of food, a rise in blood glucose levels triggers the release of insulin from pancreatic beta cells, where it acts on ‘glucose sink’ tissues, most notably skeletal muscle, in order to remove glucose from the blood. 80% of insulin-dependent blood glucose uptake is mediated by skeletal muscle cells, and a failure of this process is thought to be one of the earliest pathological events in the development of T2DM. Binding of insulin to its receptor tyrosine kinase initiates a signalling cascade (Fig. 1), the end result of which is translocation of the glucose transporter, GLUT4, to the surface of myocytes, permitting glucose uptake. The pathology of insulin resistance is complex and incompletely understood, however it is thought that, at least in part, the build-up of fatty-acid derived second messengers in muscle, adipose and liver cells inhibits the intracellular signalling pathway downstream of the insulin receptor^4–6^.

Several studies have also demonstrated that components of the insulin signalling pathway, including PTEN^7,8^ and Akt^9,10^, are subject to post-translational modification by the small ubiquitin-like modifier (SUMO, a 97 amino acid peptide), a process termed SUMOylation^11,12^. This modification is analogous to ubiquitination, with nascent SUMO peptides (paralogues SUMO1, or SUMO2/3) being first matured by SUMO-specific proteases, termed SENPs, activated by an E1 enzyme complex (consisting of SAE1 and SAE2 in mammals), and conjugated to substrates via Ubc9, the only SUMO E2 ligase. Most SUMO deconjugation is also catalysed by SENP enzymes, of which there are 6 isoforms of varying cellular distribution. SUMOylation has a well-established role in the regulation of nuclear processes, including chromosome segregation and transcriptional regulation^13^, however several recent studies have demonstrated diverse extra-nuclear roles for this modification, most notably in vesicle trafficking in neurotransmission^14–24^. With regard to the insulin signalling pathway, Akt and PTEN SUMOylation has been suggested to regulate their activity^8,9^. Furthermore, the key SUMO conjugation enzyme, Ubc9, has been shown to regulate the turnover and targeting of GLUT4 to insulin-responsive storage compartments in adipocytes^25^. There have also been several reports that SUMOylation levels and the levels of critical SUMOylation enzymes are altered in diseases such as Alzheimer’s Disease and T2DM, suggesting dysfunction of the SUMOylation system could be pathological^19,26^. Indeed, one study has demonstrated a reduction in levels of both SUMO paralogues and Ubc9 in the muscles of patients with advanced insulin resistance^27^. There is also evidence that SUMOylation is involved in fat metabolism in skeletal muscle^28,29^, providing further links between this post-translational modification and insulin resistance. Taken together, these lines of evidence present the possibility that changes in SUMOylation levels in T2DM could aberrantly affect insulin signalling and therefore contribute to skeletal muscle insulin resistance.

In order to investigate this, we manipulated SUMOylation levels in several different ways in differentiated rat L6 myocytes, using a lentiviral transduction system, and assayed the effect on insulin-dependent GLUT4 surface trafficking. To our surprise, and in contrast to all previous studies on the effect of SUMOylation on vesicle trafficking events, we found no evidence that changes in SUMOylation levels had any effect on GLUT4 trafficking to the cell surface in response to insulin stimulation. We therefore conclude that this pathway is not regulated by protein SUMOylation under the conditions we have used and, by extension, it is unlikely that alterations in protein SUMOylation play any role in the induction of skeletal muscle insulin resistance.

## Results and Discussion

### SUMOylation by SUMO1 is reduced in L6 myocytes in response to insulin stimulation

**Figure 2:**
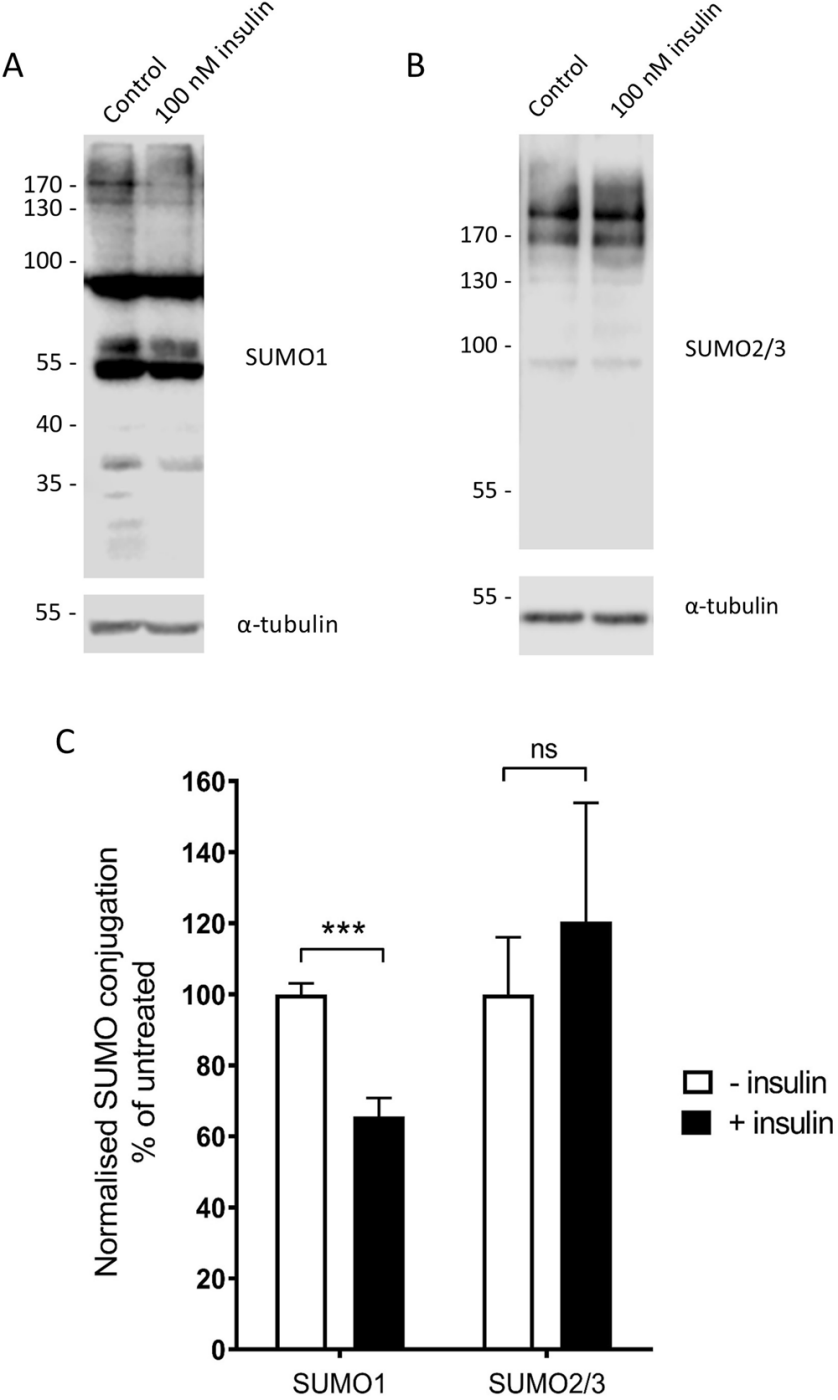
SUMOylation by SUMO1 is reduced in L6 myocytes in response to insulin stimulation. (**A**) Representative Western blot showing whole cell SUMOylation by SUMO1 in L6 myocytes treated with vehicle control or 100 nM insulin. Alpha-tubulin loading control shown below. All full-length, uncropped blots are shown in Supplementary Fig. 1. (**B**) Representative Western blot showing whole cell SUMOylation by SUMO2/3 in L6 myocytes treated with vehicle control or 100 nM insulin. Alpha-tubulin loading control shown below. (**C**) Quantification of whole cell SUMOylation by SUMO1 and SUMO2, each normalised to tubulin levels and expressed as a percentage of control SUMOylation levels. ***p < 0.001, one-sample t-test (n = 7 (SUMO1) and 6 (SUMO2/3)). Error bars = standard error of the mean.

In order to first assess whether insulin signalling in skeletal muscle cells interacts with the SUMOylation pathway, we exposed differentiated L6 myocytes to 100 nM insulin for 20 minutes, followed by cell lysis and resolution on SDS-PAGE gels. Western blot analysis was then conducted using densitometry of whole-cell SUMOylation by SUMO1 or SUMO2/3 (Fig. 2A–C). We found that insulin stimulation of differentiated L6 myocytes resulted in a robust decrease in whole cell SUMOylation by SUMO1 but not by SUMO2/3. These data therefore indicate that the insulin signalling pathway regulates SUMOylation, specifically by SUMO1, in differentiated L6 myocytes, suggesting that SUMOylation by SUMO1 potentially plays a role in the insulin signalling pathway in these cells. From results such as this it is not possible to determine which individual SUMO substrates are affected, however, since numerous SUMO1-reactive bands are decreased following insulin stimulation, these data support a general role for SUMOylation in this pathway. Consequently, we focussed on up- or down-regulation of global SUMOylation rather than interrogating the role of SUMOylation of specific candidate proteins (e.g. Akt) involved in this process.

### Overexpression of SUMO1 does not alter insulin-responsive surface expression of GLUT4

**Figure 3:**
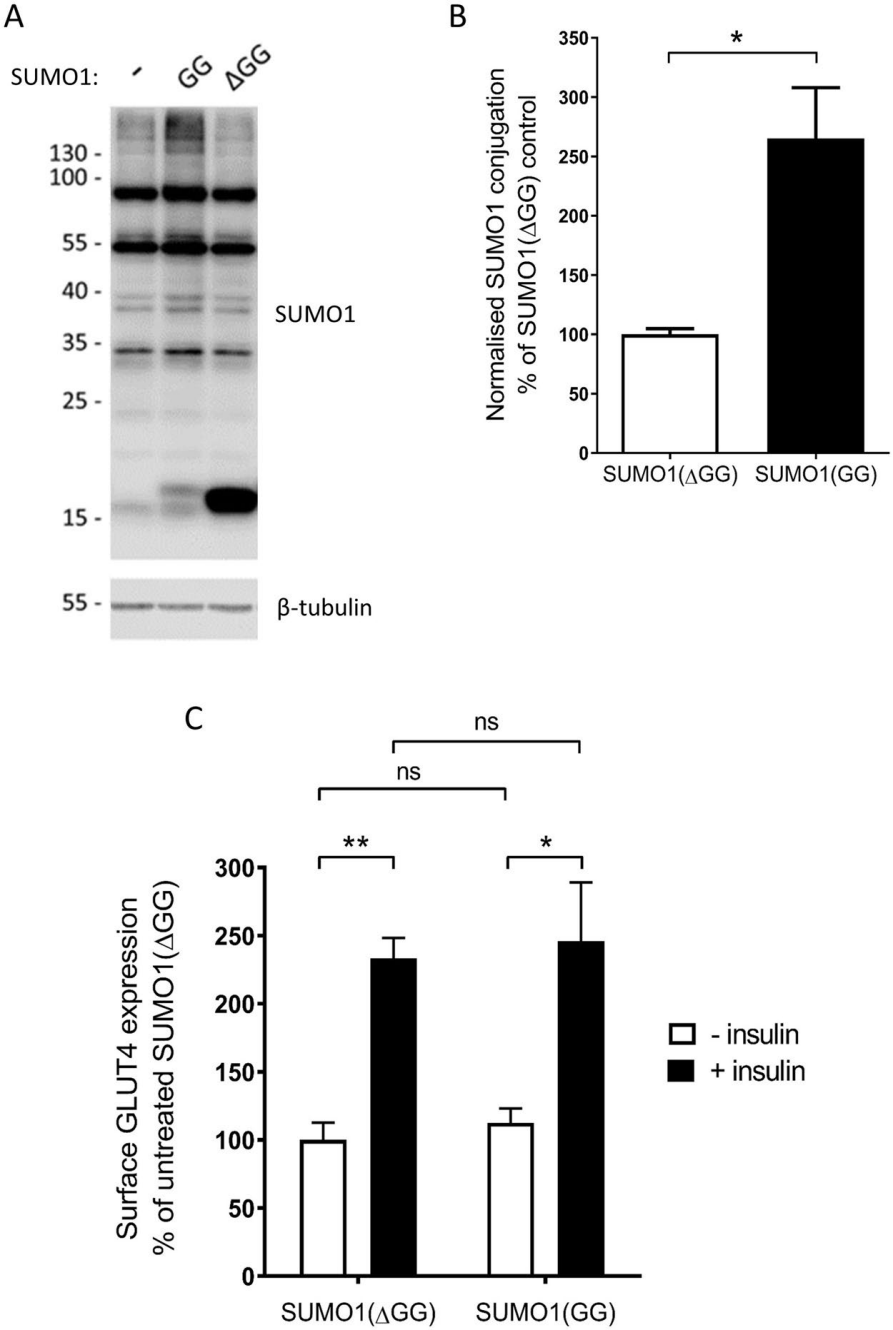
Overexpression of SUMO1 does not alter insulin-responsive surface expression of GLUT4. (**A**) Representative whole cell SUMO1 blot (above) and beta-tubulin blot (below) showing cellular SUMOylation levels in uninfected L6 myocytes and L6 myocytes infected with virus overexpressing either SUMO1(GG) or SUMO1(ΔGG). (**B**) Quantification of SUMOylation levels by SUMO1 in SUMO1(GG) or SUMO1(ΔGG) overexpressing cells, normalised to tubulin levels and expressed as a percentage of SUMOylation levels in SUMO1(ΔGG) expressing cells. *p < 0.05, one-sample t-test (n = 5). (**C**) GLUT4 surface expression assay in response to insulin in these cells, expressed as a percentage of control surface GLUT4 levels. *p < 0.05, **p < 0.01, 2-way ANOVA with Bonferroni post-hoc test (n = 5). Error bars = standard error of the mean.

In order to test the hypothesis that SUMOylation by SUMO1 regulates GLUT4 trafficking downstream of insulin signalling in L6 myocytes, we next sought to manipulate cellular SUMOylation levels by viral overexpression of SUMO1. Specifically, we overexpressed the conjugatable SUMO1(GG) peptide and SUMO1(ΔGG), which lacks the necessary diglycine motif and therefore cannot be conjugated to substrates. Western blot analysis demonstrated that overexpression of SUMO1(GG) increased total cellular SUMO1 conjugation levels (also indicating the specificity of this SUMO1 antibody), compared to the non-conjugatable SUMO1(ΔGG) (Fig. 3A,B). SUMO1(ΔGG) overexpression resulted in a dominant SUMO1-reactive band at 15 kDa, which represents free SUMO1, thus evidencing that the SUMO1ΔGG mutant cannot be conjugated to proteins. However, to our surprise, increasing total cellular SUMO1 conjugation in this manner had no measurable effect on either the basal or the insulin-responsive surface expression of GLUT4 as determined by cell surface ELISA (see Materials and Methods for details) (Fig. 3C). The insulin-induced increase in surface GLUT4 expression seen in our data is similar to that seen in previous studies using similar techniques^30^, indicating that viral infection *per se* did not affect this process. We also performed a positive control for a known SUMO1 substrate, RanGAP1^11^, by SUMO1 pulldown from L6 myocyte lysates (Supplementary Fig. 2). Affinity pulldown of SUMO1 resulted in a RanGAP1-reactive band at the predicted molecular weight of SUMOylated RanGAP1 (Supplementary Fig. 2C,D). Importantly, incubation of the L6 lysate in the absence of NEM (in order to remove SUMOylation, as NEM inhibits SENPs, the deSUMOylating proteases) resulted in a shift to the predicted molecular weight for unmodified RanGAP1, indicating that this higher molecular weight band indeed represents SUMOylated RanGAP1 (Supplementary Fig. 2A,B). A similar, negative control experiment resulted in no SUMO1 affinity pulldown of GLUT4 reactive bands (Supplementary Fig. 3), indicating that this protein is not a SUMO substrate, consistent with previous studies^25^.

Two possible explanations for these results are: (a) protein SUMOylation plays no role in the regulation of insulin-responsive GLUT4 surface trafficking in L6 myocytes; (b) overexpression of SUMO1(GG) or SUMO1(ΔGG) does not influence the SUMOylation of substrates specific to the insulin signalling pathway.

It is interesting to note that the changes in SUMOylation observed using the manipulation employed throughout this study appear to differentially affect specific bands on the SUMO ladder. This has been observed in several previous studies (e.g.^21^), however there are, as yet, no reliable methods for identifying the specific SUMOylated proteins from assays such as these.

### Overexpression of the SENP1 catalytic domain has no effect on insulin-responsive surface expression of GLUT4

**Figure 4:**
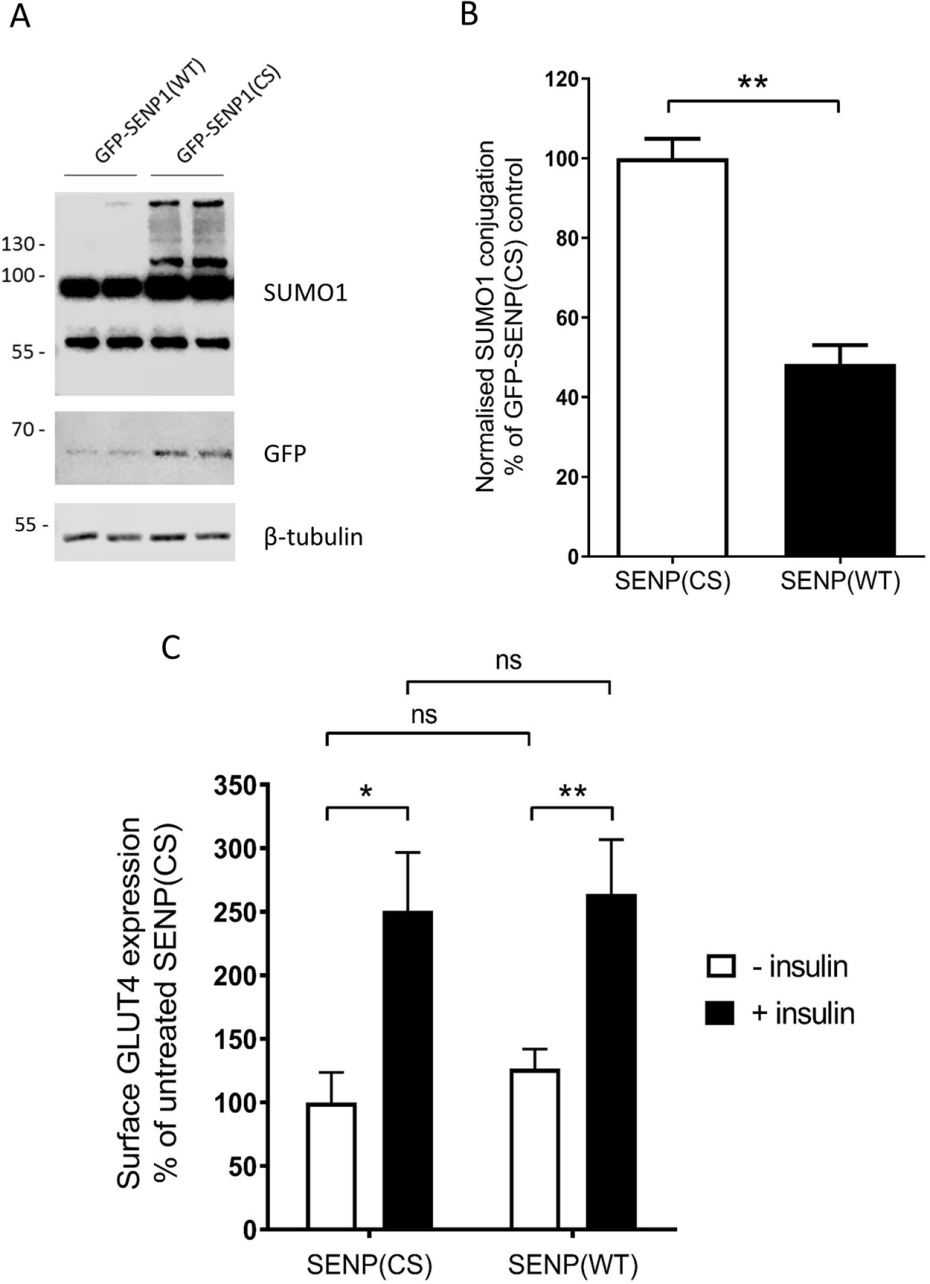
Overexpression of the SENP1 catalytic domain does not alter insulin-responsive surface expression of GLUT4. (**A**) Representative whole cell SUMO1 blot (above), GFP blot (for GFP-tagged SENP constructs) and beta-tubulin blot (below) showing cellular SUMOylation levels in L6 myocytes infected with virus overexpressing either wild type SENP1 or catalytically inactive SENP1-C603S. (**B**) Quantification of SUMOylation levels by SUMO1 in SENP1 (SENP(WT)) or SENP1-C603S (SENP(CS)) overexpressing cells, normalised to tubulin levels and expressed as a percentage of SUMOylation levels in SENP1-C603S expressing cells. **p < 0.01, one-sample t-test (n = 3). (**C**) GLUT4 surface expression assay in response to insulin in these cells, expressed as a percentage of control surface GLUT4 levels. *p < 0.05, **p < 0.01, 2-way ANOVA with Bonferroni post-hoc test (n = 4). Error bars = standard error of the mean.

In order to investigate the possibility that the methods employed in the previous experiment only resulted in changes to the SUMOylation of a subset of substrates, we sought to manipulate cellular SUMOylation levels by other means. Therefore, we used lentiviral overexpression of the catalytic domain of the deSUMOylating enzyme, SENP1. We have used this strategy in several previous studies, and demonstrated that the catalytic domain of SENP1 has a wide cellular distribution, and profoundly influences several different vesicle trafficking events^17,21,23^. Western blot analysis demonstrated a robust expression of SENP1 in infected cells, together with the expected reduction in SUMO conjugation seen in cells expressing SENP1 compared to the catalytically inactive mutant, SENP1-C603S (Fig. 4A,B). However, once again both the basal and the insulin-stimulated surface expression of GLUT4 was unaffected by the SENP1 virus when compared to the SENP1-C603S virus (Fig. 4C).

This surprising result adds further evidence that protein SUMOylation does not regulate GLUT4 trafficking to the cell surface in L6 myocytes. Furthermore, it argues against a role for SUMOylation in the regulation of the PI3-kinase-dependent insulin signalling pathway (Fig. 1). However, there is a possibility that, once again, the overexpression of the SENP1 catalytic domain fails to influence substrates specific to this process.

It is interesting to note that SENP1 overexpression results in reduction of some SUMOylated bands more than others. This is presumably indicative that some substrates are considerably more stably or heavily SUMOylated than others, for example RanGAP (at just under 100 kDa). It is likely, therefore, that proteins such as this are less susceptible to decreases in SUMOylation upon SENP1 overexpression.

### shRNA-mediated knockdown of Ubc9 does not affect basal or insulin-responsive surface expression of GLUT4

**Figure 5:**
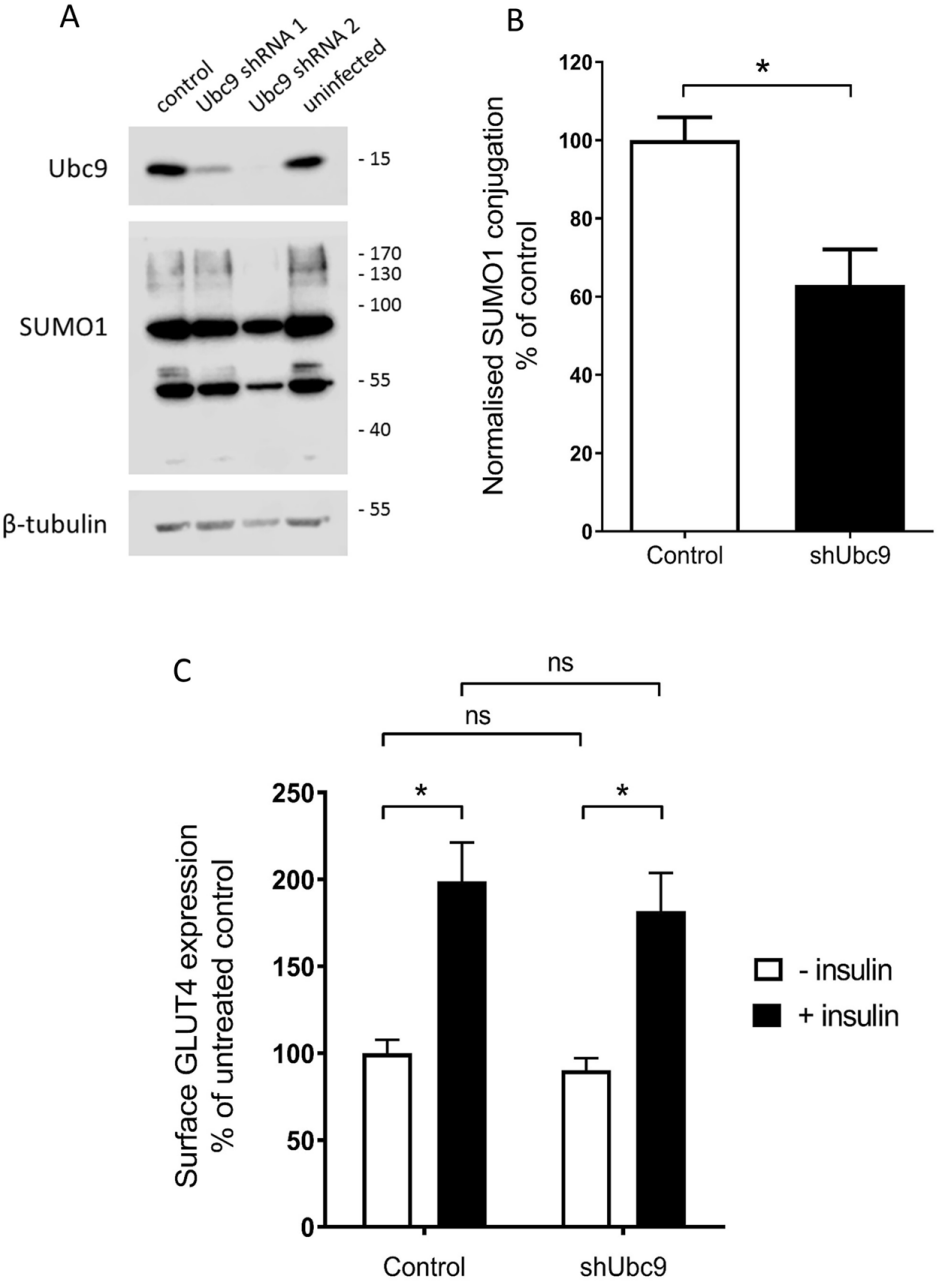
Ubc9 knockdown does not alter insulin-responsive surface expression of GLUT4. (**A**) Representative blots of Ubc9 levels (above), whole cell SUMOylation by SUMO1 (middle), and beta-tubulin (below) in L6 myocytes infected with virus expressing either scrambled (control) shRNA or one of 2 shRNA sequences directed at Ubc9. (**B**) Quantification of SUMOylation levels by SUMO1 in cells infected with virus expressing Ubc9 shRNA sequence number 2, normalised to tubulin levels and expressed as a percentage of SUMOylation levels in scrambled shRNA expressing cells. *p < 0.05, one-sample t-test (n = 4). (**C**) GLUT4 surface expression assay in response to insulin in these cells, expressed as a percentage of control surface GLUT4 levels. *p < 0.05, 2-way ANOVA with Bonferroni post-hoc test (n = 4). Error bars = standard error of the mean.

In order to determine whether the first two methods which we employed failed to affect the SUMOylation of substrates relevant to insulin signalling, we next using viral shRNA expression to reduce the expression of Ubc9, the sole SUMO E2 conjugating enzyme. Several studies have demonstrated that Ubc9 is necessary and sufficient for SUMO conjugation to substrates^31,32^, and genetic ablation of Ubc9 in mice is embryonic lethal^33^. We used two different shRNA sequences directed against Ubc9 (see Materials and Methods for details), of which sequence number 2 achieved almost complete knockdown of Ubc9 expression (Fig. 5A). Viral expression of this shRNA also significantly reduced cellular SUMO conjugation (Fig. 5A,B). We then compared both basal and insulin-responsive cell surface expression of GLUT4 in cells infected with either this virus or a scrambled shRNA control virus.

Once again, we found no differences in either basal or insulin-responsive surface expression of GLUT4 caused by this virus in comparison to the control virus (Fig 5C). These data are entirely consistent with the previous experiments. Taken together, our data demonstrate that manipulation of cellular protein SUMOylation in three different ways does not affect either basal or insulin-responsive surface expression of GLUT4. These experiments therefore strongly suggest that protein SUMOylation has no role in regulating cell surface GLUT4 expression in L6 myocytes, either under basal or insulin-stimulated conditions.

## Conclusion

In this study, we have found evidence that manipulation of protein SUMOylation does not affect the insulin-dependent surface expression of GLUT4 in differentiated rat L6 myocytes. Whilst we cannot formally exclude the possibility that SUMOylation plays a role in regulating this process, none of the strategies which we employed to manipulate SUMOylation had any effect in this system. It is possible that the strategies which we have employed did not effectively raise or lower SUMOylation levels of all substrates, however given the variety of tools we have used, we consider this unlikely. We acknowledge the possibility that these negative data are unique to this cell line or cell type. For example, a previous study has demonstrated a role for Ubc9 in GLUT4 turnover and targeting^25^, however this study was performed in adipocytes, in which the regulatory systems may be different. Additionally, we acknowledge that the high dose of insulin used in this study may mask any subtle effects of SUMOylation on insulin-dependent GLUT4 trafficking which would be apparent at lower doses of insulin. Finally, the myocytes employed in this study were not made insulin resistant, for example by treatment with high levels of saturated fatty acids. It is therefore possible that SUMOylation may play a role in the pathology of insulin resistance rather than under the ‘healthy’ conditions used in this study. This subject, therefore, warrants further investigation.

It is also possible that systems which more accurately represent human physiology would yield different results; indeed, differences in SUMOylation have been found in muscle tissue from patients with advanced T2DM, suggesting a potential role^27^. It is possible, however, that the changes seen in this study are a symptom, rather than part of the pathophysiology of T2DM, and our data support this argument. These data therefore argue against a role for changes in SUMOylation as a pathological factor in insulin resistance in T2DM, and suggest that SUMOylation does not represent a viable therapeutic target for this aspect of this disease. We fully acknowledge that our study, performed in rat myocytes, cannot be definitively extrapolated to human pathophysiology, and more research is needed in this area.

These results are therefore somewhat surprising, especially given a demonstrable effect of insulin treatment on SUMOylation by SUMO1 (see Fig. 2). It is possible that this result represents actions of insulin distinct from the surface trafficking of GLUT4, and therefore should be considered part of a different pathway or longer time-scale effects (e.g. on transcription), and further investigation in this area is warranted. It is also worth noting that this is the only vesicle trafficking event investigated so far for which the SUMOylation state of the cells appears to be irrelevant. This may perhaps reflect the mesodermal origin of muscle cells compared to the ectodermal origin of neurones and neuroendocrine cells, for which positive data have been obtained e.g.^21–24,34,35^. Indeed, there is compelling evidence from several studies that SUMOylation has a critical role in the regulation of insulin secretion from pancreatic beta cells^34–38^, implying that defective SUMOylation may yet play an important role in the pathophysiological reduction in insulin secretion seen in T2DM.

## Materials and Methods

### Cell culture

Myc-GLUT4 expressing L6 myocytes were maintained in Minimum Essential Medium Eagle - Alpha Modification (alpha-MEM) supplemented with 10% foetal-bovine serum (FBS), 2 mM glutamine and penicillin/streptomycin at 37 °C and 5% CO_2_. Myocytes were differentiated by culturing in medium containing 2% FBS for 7 days prior to fixation. Cells were infected with 20 µl appropriate lentivirus for 4 days (overexpression) or 6 days (knock-down) and subsequently assayed for insulin-induced GLUT4 surface trafficking.

### Molecular biology

To generate overexpression vectors for lentiviral production, constructs were cloned into a modified pXLG3 vector under an SFFV promoter^39^ using standard techniques. SUMO1(GG) encoded the conjugatable human SUMO1 protein after SENP maturation, whereas SUMO1(ΔGG) lacked the C-terminal diglycine motif required for conjugation^40^. GFP-tagged human SENP1 catalytic domain, and the catalytically inactive C603S mutant, have been previously described^41^. To generate Ubc9 knockdown vectors for lentiviral production, two shRNA sequences targeting rat Ubc9 (1: GCCTATACAATTTACTGCCAA, 2: TGGCACGATGAACCTGATGAA) and a non-specific shRNA control (AATTCTCCGAACGTGTCAC) were cloned into a modified pXLG3 vector under a H1 promoter using standard techniques.

### Lentiviral production and infection

Lentivirus particles were produced in HEK293T cells maintained in Dulbecco’s Modified Eagle’s Medium supplemented with 10% FBS, 2 mM glutamine and penicillin/streptomycin. HEK293T cells were co-transfected with the requisite pXLG3 vector and MISSION lentivirus packaging plasmid mix (Sigma-Aldrich) using polyethylenimine. The HEK293T media containing lentiviral particles was harvested 48 h after transfection.

### SDS-PAGE and Western Blotting

For Western blotting, cells were lysed in Laemmli buffer (2% SDS, 5% 2-β-mercaptoethanol, 5% glycerol, 62.5 mM Tris-HCl pH 6.4, 0.002% bromophenol blue), briefly sonicated to shear DNA and heated to 37 °C for 10 min. Proteins in cell lysates were separated by SDS-PAGE and transferred to nitrocellulose membrane for Western blotting. Membranes were blocked in 5% w/w non-fat milk powder in PBS-T. Primary antibodies used for blotting were: SUMO1 (rabbit monoclonal, Abcam ab133352, 1:1000), SUMO2/3 (rabbit monoclonal, Abcam ab109005, 1:1000), Ubc9 (rabbit monoclonal, Abcam ab75854, 1:3000), GFP (mouse monoclonal, Abcam ab1218, 1:500), alpha-tubulin (mouse monoclonal, Cell Signaling Technologies clone DM1A, 1:5000), RanGAP1 (rabbit monoclonal ab92360, 1:1000), beta-tubulin (mouse monoclonal, Invitrogen PAS-16863, 1:3000), myc (mouse monoclonal, Cell Signaling Technologies clone 9B11, 1:1000). For SUMO1 pulldowns, cells were lysed in 1% triton X-100, 0.1% sodium-dodecyl sulphate, 150 mM NaCl, 25 mM HEPES (pH 7.4). 20 mM NEM was either included or omitted from the lysis buffer dependent on experimental conditions. SUMO1 pulldowns were performed as previously described^24^. For protein detection, membranes were incubated with either horseradish peroxidase (HRP)-conjugated (1:10000) or IRDye-conjugated (1:20000) secondary antibodies (both from LI-COR). HRP signal was visualised by enhanced chemiluminescence, and IRDye signal by fluorescence, both using an Odyssey Fc Imager (LI-COR).

### GLUT4 translocation assay

L6 myocytes were incubated in serum-free alpha-MEM for 3 hours, and then stimulated with 100 nM insulin for 20 minutes if necessary. Cells were placed on ice, washed twice with ice-cold phosphate-buffered saline (PBS: 137 mM NaCl, 2.7 mM KCl, 10 mM Na_2_HPO_4_, 1.8 mM KH_2_PO_4_, pH 7.4) and fixed with 3% ice-cold paraformaldehyde (PFA) on ice for 10 minutes then at room temperature for a further 10 minutes. After removing the PFA, cells were washed twice with PBS and incubated with 100 mM glycine for 10 min to quench excess PFA. Cells were washed three times with PBS and blocked with 2% BSA in PBS for 15 min. To detect surface myc-GLUT4, cells were incubated with anti-myc primary antibody (mouse monoclonal, Cell Signaling Technologies clone 9B11, 1:1000 dilution in 2% BSA in PBS) at 4 °C for 1 hour with shaking. Following five PBS washes, cells were incubated with HRP-conjugated anti-mouse secondary antibody (1:1000 dilution in 2% BSA in PBS) at 4 °C for 45 minutes with shaking. For visualisation of HRP signal (surface GLUT4 expression), cells were washed five times in PBS then incubated with 3,3′,5,5′-Tetramethylbenzidine for three to five minutes. The reaction was quenched with 2 N HCl, and the absorbance at 450 nm recorded using a plate reader. Average background signal from cells in which the primary antibody step was omitted was subtracted from each well.

### Data analysis

Western blot protein band intensities were quantified by densitometry using Image Studio software (LI-COR). SUMO conjugation was quantified by densitometry of the whole lane in SUMO blots. Western blot data was normalised to the loading control, and then to the appropriate control on the same membrane, to account for differences between Western blots, resulting in all control values being 100%. Western blot data was analysed by one-sample t-test against the control value of 100%. Surface GLUT4 expression was normalised to the control condition without insulin treatment, to account for different detection thresholds between experiments, resulting in all untreated control values being 100%. In these cases, the variance of the control was calculated using the variance of the raw, non-normalised values. Surface GLUT4 expression was analysed by 2-way ANOVA using Bonferroni post-hoc tests.

## Supplementary information

Supplementary Dataset 1

## Data Availability

All data generated or analysed during this study are included in this published article.
