# Supplementary material for: Insulin-dependent GLUT4 trafficking is not regulated by protein SUMOylation in L6 myocytes
Source: Sci Rep. 2019 Apr 24;9:6477. doi: 10.1038/s41598-019-42574-3 (PMC6482176; doi:10.1038/s41598-019-42574-3)
Supplement: Supplementary file 1 — Supplementary Dataset 1 [file 41598_2019_42574_MOESM1_ESM.pdf]

Insulin-dependent GLUT4 trafficking is not regulated by protein SUMOylation in L6 myocytes

Ruth E. Carmichael[1], Kevin A. Wilkinson[2] and Tim J. Craig[1]*

[1] Centre for Research in Biosciences, University of the West of England, Coldharbour Lane, Frenchay, BS16 1QY, U.K.

[2] School of Biochemistry, Biomedical Sciences Building, University of Bristol, University Walk, Bristol, BS8 1TD, U.K.

Supplementary Information

Supplementary Fig 1. Full length blots

From Figure 2A:

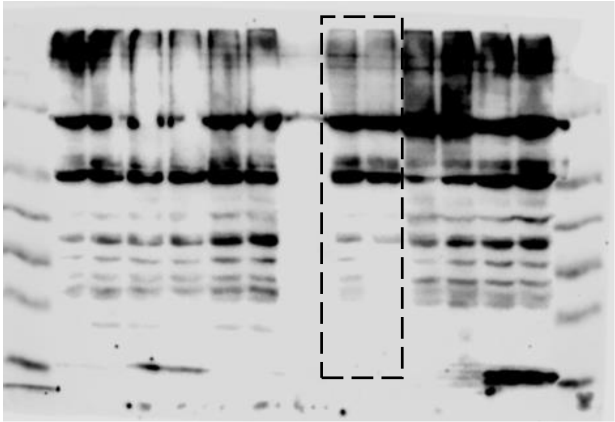

SUMO1

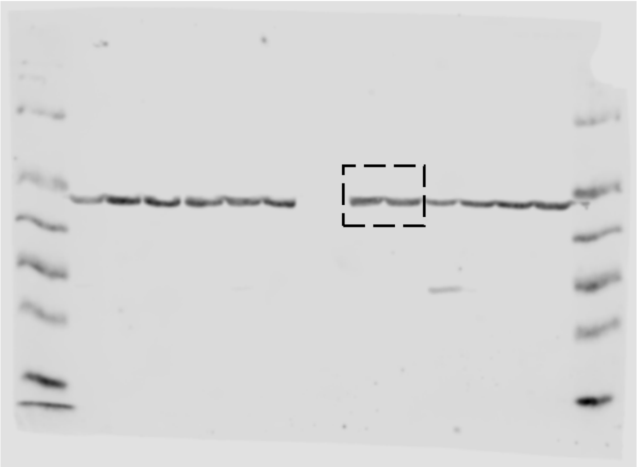

α-tubulin

From Figure 2B:

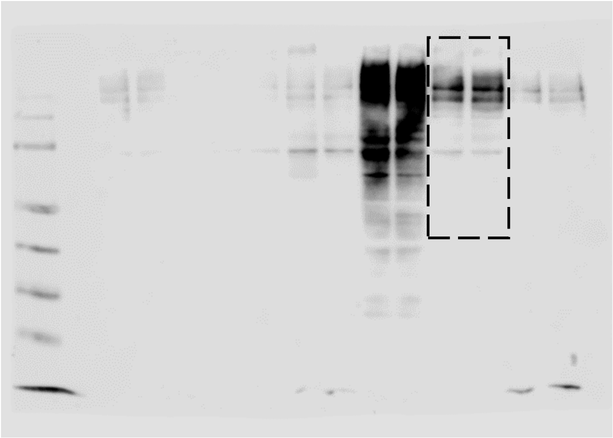

SUMO2/3

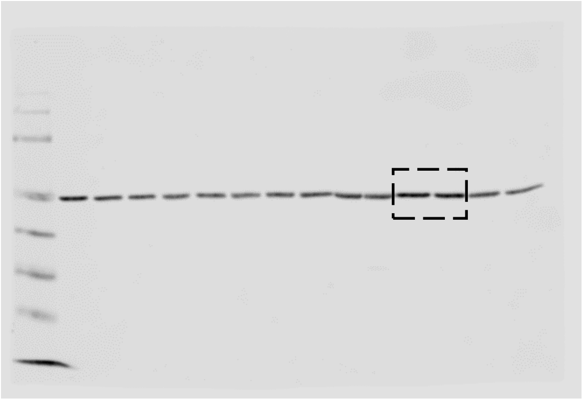

α-tubulin

<u>Supplementary Fig 1. continued</u>

From Figure 3A:

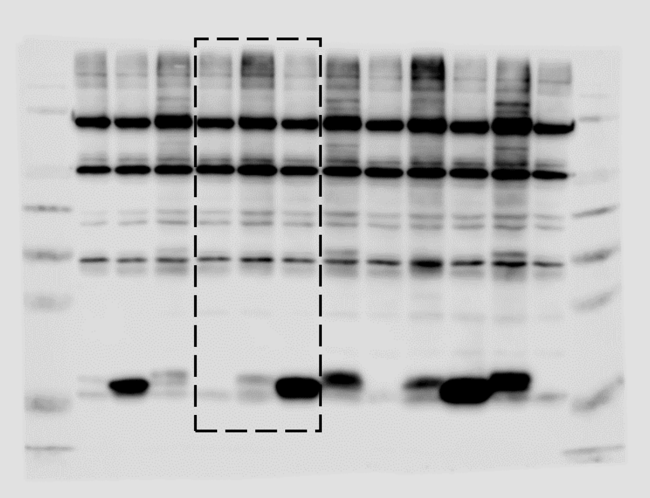

SUMO1

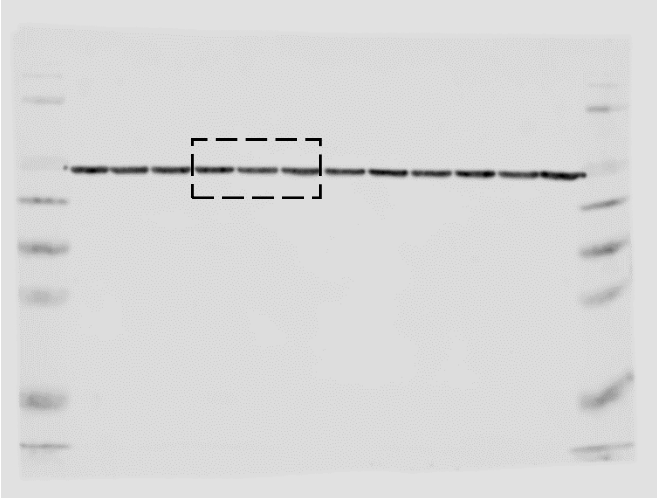

α-tubulin

From Figure 4A:

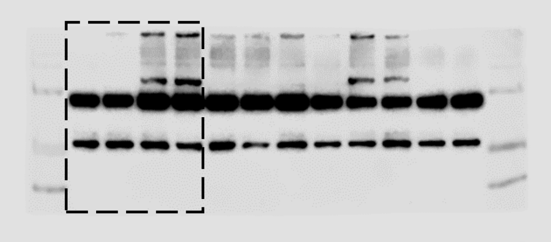

SUMO1

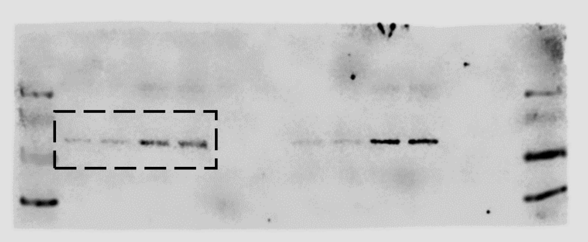

GFP

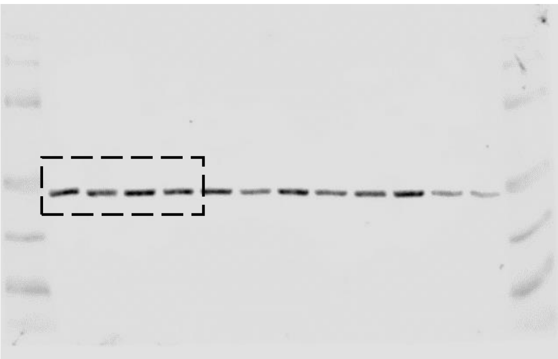

β-tubulin

Supplementary Fig 1. continued

From Figure 5A:

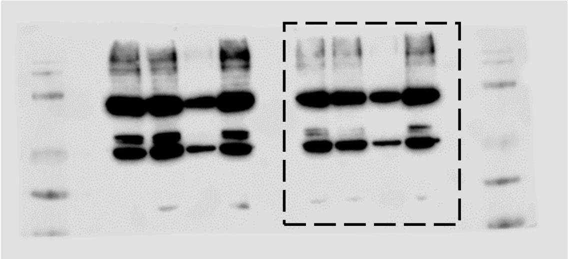

SUMO1

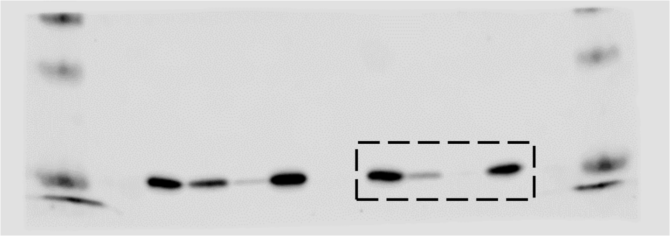

Ubc9

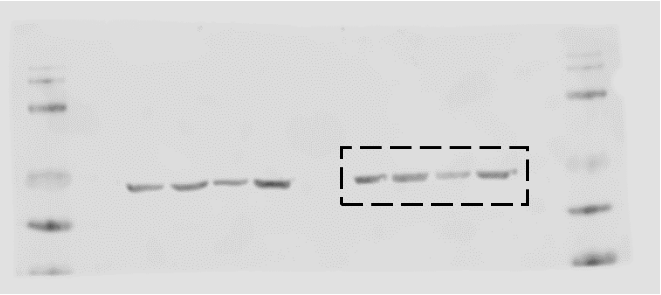

β-tubulin

Supplementary Fig 2 – Positive SUMOylation control for L6 myocytes

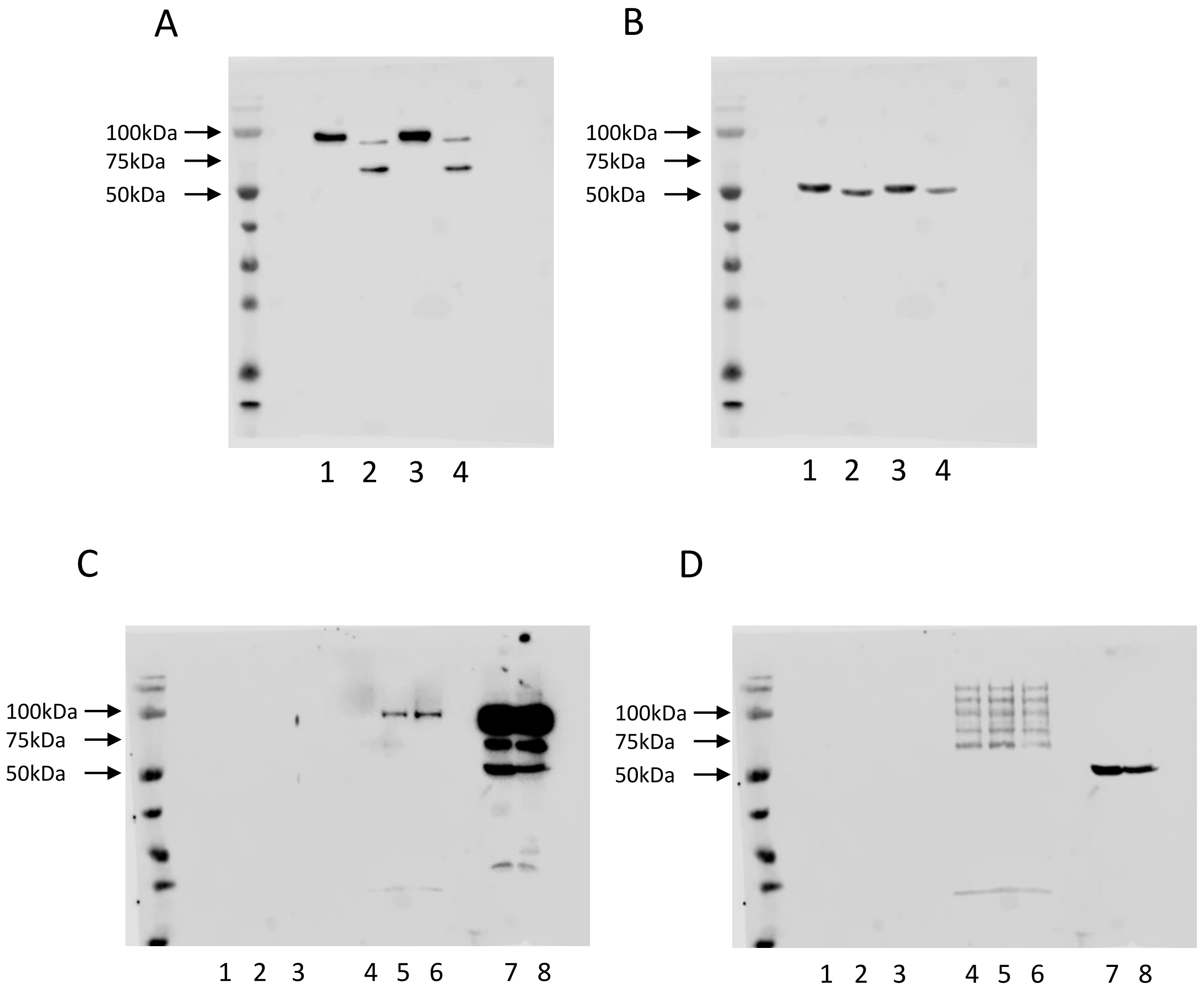

Supplementary Fig 3 – Negative SUMOylation control for L6 myocytes

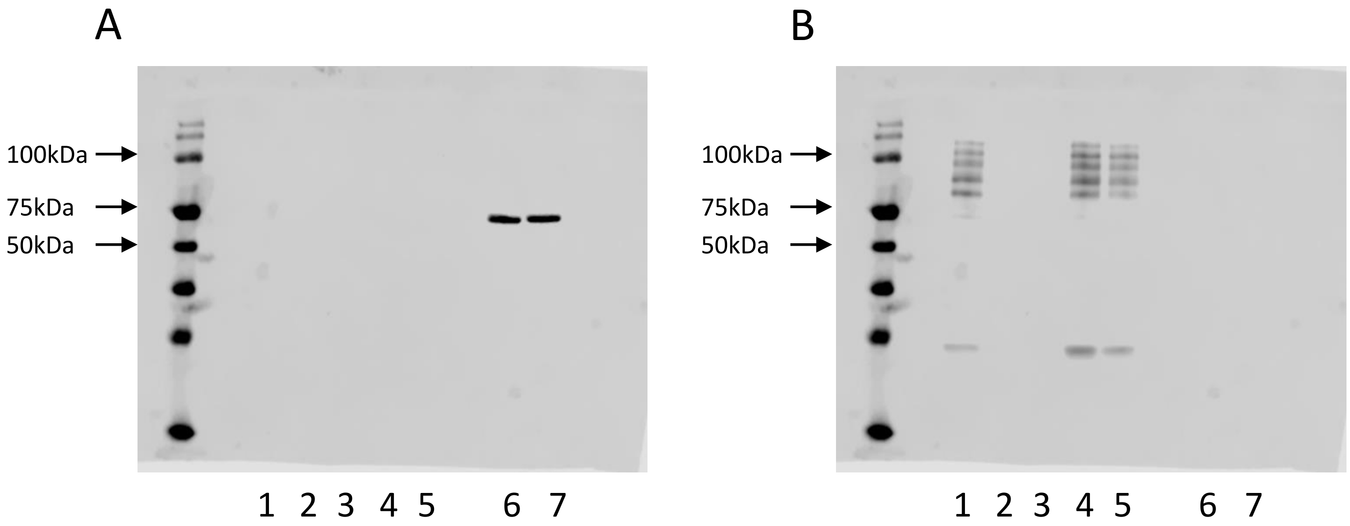

Supplementary Figure Legends

Supplementary Fig 1. Full length blots

Full length, uncropped blots for all figures. Cropped areas shown in figures are indicated with dashed boxes.

Supplementary Fig 2. Positive control for SUMOylation in L6 myocytes

A: Blot for RanGAP1 of L6 myocytes lysate treated with (lanes 1 + 3) and without (lanes 2 + 4) 20 mM NEM. **B:** Alpha-tubulin blot of A. **C:** RanGAP1 blot of SUMO1 pulldown from L6 myocytes. Lane 1 = beads with no antibody, buffer control. Lanes 2 + 3 = breads with no antibody, L6 lysate. Lane 4 = beads with antibody, buffer control. Lanes 5 + 6 = beads with antibody, L6 lysate. Lanes 7 + 8 = L6 lysate inputs. **D:** Alpha-tubulin blot of C (bands on lanes 4-6 are eluted antibody bands revealed by mouse secondary antibody).

Supplementary Fig 3. Negative control for SUMOylation in L6 myocytes

A: Blot for GLUT4 of SUMO1 pulldown from L6 myocytes. Lane 1 = beads with antibody, buffer control. Lanes 2 + 3 = beads with no antibody, L6 lysate. Lanes 4 + 5 = beads with antibody, L6 lysate. Lanes 6 + 7 = L6 lysate inputs. **B:** Mouse secondary antibody blot of A to reveal antibody bands.
